# Brugada Syndrome: From Molecular Mechanisms and Genetics to Risk Stratification

**DOI:** 10.3390/ijms24043328

**Published:** 2023-02-07

**Authors:** Irene Paula Popa, Dragomir N. Șerban, Minela Aida Mărănducă, Ionela Lăcrămioara Șerban, Bogdan Ionel Tamba, Ionuț Tudorancea

**Affiliations:** 1 Cardiology Clinic, “St. Spiridon” County Clinical Emergency Hospital, 700111 Iași, Romania; 2 Department of Physiology, “Grigore T. Popa” University of Medicine and Pharmacy, 700115 Iași, Romania; 3 Department of Pharmacology, “Grigore T. Popa” University of Medicine and Pharmacy, 700115 Iași, Romania

**Keywords:** Brugada syndrome, arrhythmias, sudden cardiac death, genetics, mutations, *SCN5A*, loci, susceptibility, genome-wide association, molecular mechanism

## Abstract

Brugada syndrome (BrS) is a rare hereditary arrhythmia disorder, with a distinctive ECG pattern, correlated with an increased risk of ventricular arrhythmias and sudden cardiac death (SCD) in young adults. BrS is a complex entity in terms of mechanisms, genetics, diagnosis, arrhythmia risk stratification, and management. The main electrophysiological mechanism of BrS requires further research, with prevailing theories centered on aberrant repolarization, depolarization, and current-load match. Computational modelling, pre-clinical, and clinical research show that BrS molecular anomalies result in excitation wavelength (k) modifications, which eventually increase the risk of arrhythmia. Although a mutation in the *SCN5A* (Sodium Voltage-Gated Channel Alpha Subunit 5) gene was first reported almost two decades ago, BrS is still currently regarded as a Mendelian condition inherited in an autosomal dominant manner with incomplete penetrance, despite the recent developments in the field of genetics and the latest hypothesis of additional inheritance pathways proposing a more complex mode of inheritance. In spite of the extensive use of the next-generation sequencing (NGS) technique with high coverage, genetics remains unexplained in a number of clinically confirmed cases. Except for the *SCN5A* which encodes the cardiac sodium channel NaV1.5, susceptibility genes remain mostly unidentified. The predominance of cardiac transcription factor loci suggests that transcriptional regulation is essential to the Brugada syndrome’s pathogenesis. It appears that BrS is a multifactorial disease, which is influenced by several loci, each of which is affected by the environment. The primary challenge in individuals with a BrS type 1 ECG is to identify those who are at risk for sudden death, researchers propose the use of a multiparametric clinical and instrumental strategy for risk stratification. The aim of this review is to summarize the latest findings addressing the genetic architecture of BrS and to provide novel perspectives into its molecular underpinnings and novel models of risk stratification.

## 1. Introduction

Brugada syndrome (BrS) is a relatively rare hereditary arrhythmia disorder that was initially documented in 1992 [1]. Despite the latest hypothesis of additional inheritance pathways, BrS is still considered a Mendelian condition inherited in an autosomal dominant manner [2]. It is considered responsible for 4% of all sudden cardiac death (SCD) and nearly 20% of SCD in individuals with structurally normal hearts [3]. Arrhythmias, such as ventricular tachycardia (VT), ventricular fibrillation (VF), bradycardia, or atrioventricular node conduction dysfunction, are common in patients with BrS, with males being more prone than females to experience these heart rhythm disturbances [4]. BrS was previously presumed to be a primary electrical disorder of the structurally normal heart (6,7), specifically, an idiopathic VF produced by aberrant electrophysiologic activity in the right ventricular epicardium [5]. Nevertheless, structural anomalies in patients’ right ventricle outflow tract (RVOT) imply that Brugada syndrome may be part of a broader spectrum of right ventricular cardiomyopathies [6]. The main electrophysiological mechanism of BrS requires further research, with prevailing theories centered on aberrant repolarization, depolarization, and current-load match. Computational modelling, pre-clinical, and clinical research show that BrS molecular anomalies result in excitation wavelength (k) alterations, which eventually increase the risk of arrhythmia (10). Genetic variants have been discovered in 11–28% of Brugada syndrome patients, with a significant percentage involving the *SCN5A* (Sodium Voltage-Gated Channel Alpha Subunit 5) gene [7]. Several different genes have been postulated to induce BrS, but their implications are strongly contested [8,9]. Understanding the genetic aetiology of BrS is essential for determining asymptomatic genetic carriers at risk for SCD and stratifying the probability of future arrhythmic incidents [10]. The lack of a connection between genotype and phenotype in BrS-affected families emphasizes the complexity of this arrhythmogenic disorder and indicates that the onset and prognosis of BrS are probably a consequence of a mixture of various gene mutations and external conditions encompassing a delicate balance of ion currents [11,12]. The primary challenge in individuals with a BrS type 1 ECG is to identify those who are at risk for sudden death, researchers proposing the use of a multiparametric clinical and instrumental strategy for risk stratification.

## 2. Clinical Characteristics and Diagnostic Criteria of BrS

BrS is commonly diagnosed during the fifth decade of life, with an average age of 41 ± 15 years; however, it has also been reported in pediatric and geriatric patients [13]. Although many asymptomatic individuals with a Brugada ECG are fortuitously discovered, a minority of patients with BrS experience symptoms, such as nocturnal agonal breathing, syncope, and/or palpitations, which may progress to ventricular arrhythmias or SCD [13]. Supraventricular arrhythmias, including atrial fibrillation, atrial flutter, AV nodal re-entry tachycardia (AVNRT), and pre-excitation syndromes (Wolf–Parkinson–White syndrome) [14], may occur in up to 20% of BrS patients. In relation to the condition, increased sinus node recovery time and sino-atrial conduction time [15] together with delayed atrial conduction and atrial standstill have been described [16]. The development of supraventricular arrhythmias is strongly linked with ventricular inducibility. Among the Brugada syndrome patients with an indication for an implantable cardioverter-defibrillator (ICD) 27% exhibit supraventricular arrhythmias, compared to 13% of patients without an ICD indication, suggesting a more severe disease course in patients with spontaneous atrial arrhythmias [17].

After the initial expert consensus from 2002 regarding the diagnostic criteria for BrS [18], updates have been made in successive reports released in 2005 [19] and 2013 [20], along with technical electrocardiogram (ECG) standards in 2012 [21] and treatment guidelines in 2017 [22]. The latest expert consensus assessment, published in 2016 [23], introduced the Shanghai Score System, which assigns points based on ECG characteristics, clinical and family history, and genetic results. Up until now, the accuracy of the Shanghai Score System has been evaluated by one single-center retrospective study [24], which indicated that malignant cardiac events exclusively appeared in patients with scores that met diagnostic criteria. A type 1 ECG spontaneously occurring or being in a febrile state or following a drug provocation test, together with a Shanghai Score of 3.5 points, is necessary for the diagnosis of BrS. Despite a Shanghai Score of 3.5 in the lack of a type 1 ECG, BrS is not diagnosed. Nevertheless, it is considered that a type 1 ECG solely, whether spontaneous or documented during a provocation test, is positive for BrS. Given the relative difficulty and absence of supporting evidence in its prognostic value, the Shanghai Score System has not yet achieved universal recognition; therefore, a large percentage of medical centers continue to diagnose BrS merely on a type 1 ECG in the absence of particular adjustable variables [25].

There are three Brugada ECG patterns described: type 1 ECG is characterized by a coved ST elevation of ≥2 mm in V1-V3, followed by negative T waves, type 2 ECG has >2 mm of saddleback-shaped ST elevation, and type 3 has the morphology of either type 1 or type 2, but with <2 mm of ST-segment elevation [26]. Sodium channel blockers, such as ajmaline, procainamide, flecainide, disopyramide, propafenone, and pilsicainide, are beneficial in diagnosing BrS in circumstances when ST-segment elevation is not pathognomonic under baseline instances [27,28,29,30]. A negative I_Na_-block test does not, however, rule out a latent type of BrS (for example, the negative predictive value of the flecainide test is 36%) [30,31]. Ajmaline and pilsicainide appear to be far more efficient than flecainide [32] or procainamide [28] in uncovering BrS. Brugada syndrome might also be revealed by bradycardia, a fever condition, or vagotonic drugs [27,33,34,35,36]. Positioning the right precordial leads at a higher position (one or two intercostal spaces above standard) might improve the sensitivity of the ECG for identifying the Brugada phenotype in certain individuals, both with and without a drug provocation test [37,38,39]. Various acute and chronic disorders can cause an ECG morphology mimicking BrS, which needs to be differentiated from Brugada syndrome. Among these, acute coronary events, pericarditis, myocarditis, pulmonary embolism, metabolic diseases, altered ion balance, dissecting aortic aneurysm, thiamine deficit, electric shock, and several drugs are the most frequently reported acute conditions [40]. Left ventricular hypertrophy, athlete’s heart, right bundle branch block (RBBB), pectus excavatum, septal hypertrophy, arrhythmogenic right ventricular cardiomyopathy/dysplasia (ARVC/D), autonomic nervous system abnormalities, Duchenne-dystrophy, Friedreich’s ataxia, mediastinal tumor, and Chagas disease are among the most frequently reported persistent causes mimicking the ECG morphology of Brs [21,41].

## 3. Pathophysiology of BrS

The need for a better comprehension of pathophysiological processes in BrS stems from the unanswered concerns about diagnosis, risk stratification for arrhythmia, and treatment [42]. The three prevailing electrophysiological theories behind BrS are centered on altered depolarization, repolarization, and mismatched current load [26].

### 3.1. Depolarization Theory

The conduction velocity (CV) of propagating cardiac action potentials (AP) includes both sodium channel activation resulting in cellular depolarization and gap junction conduction throughout subsequent cardiomyocytes. Any impairment to the adequate formation or propagation of action potentials may result in conduction abnormalities and arrhythmogenesis [43]. Almost one-fourth of BrS cases have been linked to genetic variants in the *SCN5A* gene that result in a reduced inward current during phase 0 [44]. It has been established that the slower upstroke during phase 0 and the subsequent delay in action potential formation have a significant impact on ventricular arrhythmogenesis in BrS. Martini and colleagues reported fibrotic lesions in the right ventricles, which may explain the right bundle branch block and the ST segment elevation on the electrocardiogram [45]. Several research addressing the disruption of *SCN5A* in animal models have indicated that mice with a targeted disruption of *SCN5A* had a lower conduction velocity in interstitial fibrosis [46,47,48]. 

There is a strong correlation between ECG phenotype and RVOT abnormalities. In individuals with BrS, an aberrant delayed potential was measured from the epicardium of the RVOT via an electrode placed into the conus branch of the right coronary artery (RCA) [49]. Furthermore, patients with a normal ECG at baseline may have BrS-type ECG characteristics during an acute myocardial infarction involving the right coronary artery [50]. Recent research that studied panoramic ventricular mapping in humans revealed electrogram lengthening and fragmentation, implying a decreased conduction velocity and an increased scattering [51]. According to the subtype, abnormal depolarization may influence the pathophysiology to various degrees. In instances of BrS when impaired calcium or potassium function is detected, repolarization anomalies might have a major impact, since the currents regulated by these channels play an important role in the plateau phase of the cardiac action potential instead of the depolarizing period [26].

### 3.2. Repolarization Theory

According to the repolarization hypothesis, the BrS phenotype is mainly caused by variable action potential duration (APD) shortening throughout the myocardial wall. Loss-of-function *SCN5A* genetic variants may have antagonistic effects on the rapid and delayed inactivation of Na^+^ channels, leading to various repolarization outcomes [52]. At slow cardiac rates, disturbances in rapid inactivation induce a persistent Na^+^ current, which lengthens repolarization. However, the intermediate kinetic component of slow inactivation is increased, delaying Na^+^ channel restoration, decreasing Na^+^ current, and shortening APD at high heart rates. In panoramic mapping research on BrS patients, the same biphasic pattern of APD, consisting of delaying succeeded by shortening, was described [51]. APD measured values from the epicardium (particularly the RVOT epicardium) were shorter than those acquired from the endocardium attributable to a considerably higher transient outward current in the epicardium (Ito). This is demonstrated by the more pronounced loss of dome-shaped AP morphology observed in the epicardium. Originally postulated by Yan and Antzelevitch in 1999, this is considered to explain re-entry through a phase 2 re-entrant pathway. Phase 2 re-entry involves electrotonic interactions and the diffusion of epicardial sites with an AP dome to sites in which this dome is lacking [53]. This might be the culprit of the R-on-T phenomenon, which results in an extrasystolic action potential prone to triggering ventricular arrhythmias [54]. ECG ST segment elevation and T-wave inversion are induced by rapid and inverse repolarization gradients. 

Elevated Tpeak–Tend, a repolarization ECG indicator, is present in BrS patients and is related to a greater risk of arrhythmias or sudden cardiac death [55]. In addition, electrogram readings have shown a mixture of high repolarization gradient and delayed repolarization at the RVOT [51]. Ventricular tachycardia and ventricular fibrillation are probably triggered by impaired repolarization in individuals with BrS owing to calcium or potassium current imbalances. In addition, a re-entrant mechanism has been hypothesized as a consequence of Ca^2+^ channel mutations that lead to a loss of function [56]. Furthermore, the restoration theory suggests that a slope of the APD restitution curve greater than one is responsible for the appearance of repolarization alternans. APD alternans may generate high gradients in repolarization and refractoriness, as well as unidirectional conduction block and re-entry [57]. 

### 3.3. Current-Load-Mismatch, Depolarization-Repolarization Balance and Excitation Wavelength (λ)

In 2010, Hoogendijk and colleagues proposed the current-to-load mismatch subepicardial phenomena as the source of ventricular arrhythmias in individuals with BrS. Subepicardial excitation failure or deferred activation by current-to-load mismatch was discovered to be caused by a decrease in sodium current owing to channel malfunction or pore size. Computational model simulations also revealed that a disturbance in the homeostasis between the inward and outward currents might influence excitation, resulting in consequent ST-segment elevation [58,59]. In an explanted human heart model, it was shown that only the lack of local excitation was connected with ST-elevation, and not deferred activation or early repolarization [60]. Thus, by adjusting the I_to_ or I_Ca_ to adapt to the decreased sodium current, the degree of ST-elevation will be lowered [58]. To replicate comparable ST-elevation circumstances in pseudo-ECG recordings, conduction block, and excitatory failure were triggered by inhibiting sodium channels with ajmaline [59].

BrS patients have cardiac structural anomalies, particularly in the right ventricle (RV) and RVOT, which contribute to current-load-mismatch and excitation failure. This was subsequently validated by Ten Sande and colleagues through cardiac activation mapping, demonstrating that anatomical abnormalities in the subepicardial of the RV and RVOT are the most probable source of conduction alterations and ST-segment elevation [61]. This structural–electrophysiological connection is consistent with the presence of ventricular arrhythmias in BrS patients in their thirties when cardiac interstitial fibrosis is more pronounced [62]. Other research hypothesized that current-to-load mismatches at discontinuities might cause a level of conduction block, which would account for the RBBB morphology seen in BrS patients. Additionally, it should be emphasized that discontinuities are often related to depolarization anomalies in BrS arrhythmias [63]. This also interferes with action potential repolarization and recovery to produce the excitation (λ) determined by the product of effective refractory period (ERP) and conduction velocity. Both in pre-clinical animal models and BrS patients, a lower λ value has been linked with a higher probability of re-entrant arrhythmias [64,65].

**Figure 1:**
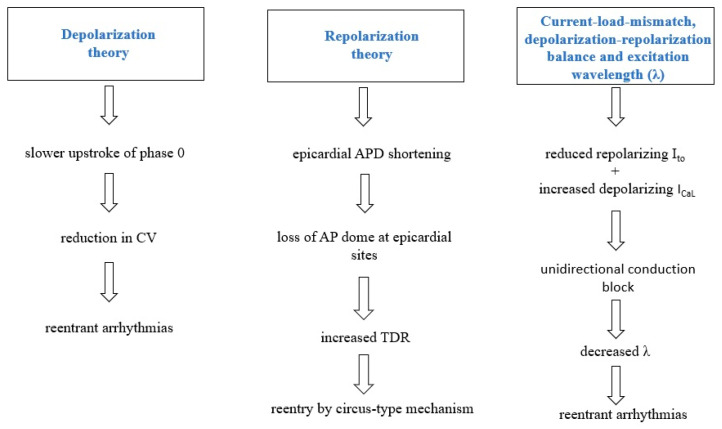
Schematic representation of the three electrophysiological theories behind BrS; AP (action potential); APD (action potential duration); CV (conduction delay); I_to_ (transient outward potassium current); I_CaL_ (L-type Ca^2+^ current); TDR (transmural dispersion of repolarization).

Various sources of evidence support each of the postulated Brugada syndrome mechanisms, as most disorders arise and manifest via many processes. Although there is research that supports the different theories discussed previously, the far more convincing evidence is derived from human research of the heart and supports the repolarization theory. Ischemia-based models are derived from depolarization models; however, ischemia does not directly contribute to the condition. Furthermore, models for the depolarization theory illustrate the repolarization hypothesis. Arrhythmia and mutations in the Na+, K+ and Ca2+ channels are compatible with the repolarization theory. Research that modifies the Na+ channels does not give credence to the depolarization theory [66]. Excitation failure by current-to-load mismatch induces ST-segment elevation, which is regulated by INa, Ito, and ICaL, comparable to ST-segment elevation in Brugada patients. Thus, the current-to-load mismatch could explain the pathophysiology behind Brugada syndrome [59]. The three electrophysiological theories behind BrS are summarized in Figure 1.

## 4. Genetics of BrS

Following the initial discovery of two symptomatic siblings in 1992, familial inheritance was hypothesized based on the original description of the BrS [1]. Based on SCD and persistent ST elevation within a two-generation family, Kobayashi et al. validated the inheritance of the condition [67]. Multiple studies further confirmed the genetic basis of BrS [68,69,70]. Probst and collaborators investigated 13 families with BrS-ECGs+ in which, at least five family members had the hereditary *SCN5A* variant. This correlation was found to be challenging, as BrS-ECGs+ were identified in just 18% of mutation carriers at baseline and in 61% following drug testing, while 8 participants exhibited BrS-ECGs+ but did not carry the familial variant. These data may indicate that the presence of an *SCN5A* mutation is not a determinant of BrS-ECG+ occurrence. The genetic background provides varying susceptibilities to the impact of a single sodium channel loss-of-function mutation, being potent enough to induce a BrS-ECG+ irrespective of an *SCN5A* mutation [70]. Despite the fact that genetics remain unidentified in a number of clinically proven cases, irrespective of the extensive implementation of next-generation sequencing (NGS) with high coverage (at least 100×), BrS is still regarded as a genetic condition [71]. Genetic variants in at least 26 distinct genes have priorly been assumed to be responsible [72]; however, the deleterious role of all genes except *SCN5A* has lately been contested [8], several genetic variants, such as polymorphisms and non-genetic variants, including fever, are being considered to influence the disease’s expression [73]. In addition, numerous genetic tests that identify genetic variants in these genes provide findings of unclear relevance. The basis of this ambiguity is frequently the assumption that all instances of BrS are inherited via an autosomal dominant Mendelian pathway. This concept limits the geneticist from evaluating a potential cumulative effect from both common and rare genetic variants since there can only be one mutation based on the prior premise. Therefore, the importance of cumulative genetic variants within a single person in the underlying disease manifestation is a topic of consideration [74]. Various genetic variants within a gene might be accountable for a variety of phenotypes [75], even within the same family [76,77], making connections between genotype and phenotype particularly challenging.

*Sodium channel mutations.* The first genetic mutation related to BrS was described in 1998 [7] regarding the sodium channel protein type V subunit α gene (*SCN5A*), which encodes the alpha subunit of the voltage-gated NaV1.5 cardiac sodium channel, responsible for the modulation of the rapid sodium current in myocytes. Ever since, around 500 sporadic mutations in more than 40 genes have been described as presumably being related to BrS. These genes mainly encode sodium, potassium, and calcium channels, as well as proteins related to them [78]. The discovery of additional genes that may be linked with BrS has allowed genetic screening in clinical assessment. Nonetheless, an extensive study of all probable BrS-associated genes revealed a disorder mutation in less than 40% of confirmed cases, keeping the genetic origin of the condition undiscovered in the vast majority of families [79]. Currently, *SCN5A* is the primary gene linked with BrS, and roughly 30% of documented cases are imputable to one of the gene’s more than 350 genetic variants [80]. Copy number variations (CNVs) are further infrequent mutations which might induce BrS. Less than ten CNVs possibly related to BrS have been identified so far, and they are all found in the *SCN5A* gene [81,82,83]. In 2011, Eastaugh et al. described the case of the first BrS patient with a significant rearrangement involving the deletion of exons 9 and 10 of *SCN5A*. Standard sequencing omitted this rearrangement, which was uncovered via a quantitative method. Additionally, it was hypothesized that it would lead to haploinsufficiency. These results prompted the researchers to propose that the analysis of CNVs in *SCN5A* be included as a regular feature of genetic testing in BrS patients [81]. According to Mademont–Soler et al. in the largest screening conducted in 2016 for CNVs in *SCN5A* in genotype-negative BrS patients and assessment of their prevalence in BrS-associated minor genes, even though such rearrangements in *SCN5A* are not particularly widespread among BrS patients, they are identified at least in several of the instances who test negative for disease-causing variants in BrS-related genes utilizing standard sequencing techniques [82]. 

A plethora of studies have investigated the possibility of modulation of *SCN5A* genetic variants by common polymorphisms [84]. A genetic component which connects all *SCN5A*-related cases remains to be identified, presumably because BrS is a distinctive ECG pattern induced by several variables and not a singular mutation, a theory based on the observation that sodium channel blockers do not induce the BrS ECG pattern in healthy persons or all *SCN5A* mutation carriers [60]. In a prior genome-wide association study (GWAS) performed on 312 patients with BrS, three common susceptibility variants were discovered, with two alleles on chromosome 3 and one allele on chromosome 6 located near the genes *SCN5A*, *SCN10A*, and *HEY2*, thus indicating an intricate genetic architecture. Results suggested that susceptibility to BrS may be caused by common genetic variations in sodium channel genes or the transcriptional regulator *HEY2* [74]. One study analyzed the connection between polygenic risk scores, which are considered predictive indicators for the outcomes of ajmaline testing, and the decelerating of cardiac conduction induced by ajmaline. The premise of the study was that a weighted mixture of certain prevalent genetic variations forecasts the individual reaction to sodium-channel blockage. The research established that rare coding variants in *SCN5A* and common non-coding variants in the *SCN5A*–*SCN10A* locus impact not only the baseline ECG, but additionally the cardiac electrical response to sodium-channel blockage. The research outcomes may have relevance in two clinical situations: single-nucleotide polymorphisms (SNP) genotyping can be utilized to determine pre-test likelihood when conducting drug testing in individuals suspected of having BrS and genotyping ahead to the prescription of sodium-channel blocking action medications. Consequently, it is necessary to acknowledge that the significance of common variations in disease susceptibility is far more extensive than initially assumed [85].

In a comprehensive genome-wide association meta-analysis including 2820 unrelated BrS cases and 10,001 controls, a number of 12 loci (10 new) were reported as being correlated with BrS. The eight independent association signals at the *SCN5A–SCN10A* locus indicate the significance of impaired sodium channel activity in the BrS’s susceptibility, whilst the eight loci containing cardiac transcription factor genes imply transcriptional regulation as a major component of BrS’s pathogenesis [86]. In BrS pathogenesis, functional analyses of MAPRE2 indicate a novel mechanism of NaV1.5 regulation through the microtubule network. The UK Biobank findings reveal a genetic overlap among the BrS and cardiac electrical characteristics and prevalent diseases in the overall population. Polygenic risk score results reinforce the theory that the disorder threshold in BrS patients is attained by various contributions of rare *SCN5A* genetic variants, common risk alleles, and sodium channel inhibition [86].

The latest research has been centered on identifying the phenotypic consequences of various polymorphisms within the *SCN5A* gene [72,75,87,88,89,90,91,92] and searching for other genes implicated in this multi-causal disease [80,90,93]. One such study established the similarities in phenotype amongst individuals with *SCN10A* genetic variants, in comparison with *SCN5A* genetic variants, which included a personal history of cardiac arrest/syncope, spontaneous BrS ECG pattern, family history of sudden death, and arrhythmic substrate [90]. This is compatible with functional research conducted on human-induced pluripotent stem cell-derived cardiomyocytes, wherein single-cell phenotypic characteristics of BrS were detected in cells from a patient with *SCN10A* gene variations [94]. The results of a multicenter analysis in which potential genes, including *SCN10A*, were sequenced did not indicate a significant involvement for *SCN10A* variations as monogenic aetiology of BrS [95]. Nevertheless, research by Hu and colleagues revealed *SCN10A* as a key susceptibility gene for BrS, detecting *SCN10A* genetic variants in 25 of 150 probands (17%), indicating that this gene plays a significant part in BrS [96]. Research on the effects of the *SCN10A* gene on cardiac conduction [97] and the autonomic nervous system [98] highlights the significance of this gene. Despite their function being debatable, the sodium channel genes *SCN1B*, *SCN2B*, and *SCN3B* have been linked to BrS and are present in the BrS diagnostic panels [8].

*Non-coding variants.* The functional impact of variants in coding areas is simple to estimate, given that the genetic code is already identified, a substantial quantity of exome data is accessible, and a thorough understanding of Mendelian illnesses exists [99]. Nevertheless, it is challenging to estimate the impact of variants in non-coding areas. These noncoding variants are present in cis-regulatory elements (CREs), including enhancers, promoters, and insulators, in addition to areas which encode non-coding RNAs (ncRNAs) [100]. A significant percentage of non-coding variants associated with cardiovascular illnesses or traits have been identified through GWAS [101]. GWAS performed on ECG traits and cardiac conduction abnormalities have emphasized the connection between the *SCN5A–SCN10A* locus and QRS duration and PR interval, indicating a role in cardiac conduction and heart function [97,102]. Notably, the majority of variants detected in this research are found in non-coding areas. It is well established that *SCN5A* variants are correlated to cardiac arrhythmias. Nevertheless, the discovery of *SCN10A* as a substantial risk area for ECG traits proved noteworthy [103,104,105]. *SCN10A* encodes the alpha subunit of the sodium channel Nav1.8, which participates in cardiac electrophysiology; however, the molecular basis continues to remain debatable, particularly in terms of its expression in cardiomyocytes [106]. Several studies have established that NaV1.8 participates in the late cardiac sodium current [107,108] and that it may be relevant in arrhythmogenesis [109]. Nonetheless, a recent study revealed that rabbit and hiPSC-CM cardiac cells lack functional NaV1.8 channels [110]. Van den Boogaard and colleagues’ research identified an additional potential function of *SCN10A* non-coding variants in conduction abnormalities. Specifically, they demonstrated that the *SCN10A* intronic variation rs6801957, which was found in a GWAS as being related to cardiac conduction dysfunction, is localized in an enhancer area that regulates the expression of the *SCN5A* gene [111,112]. Through the investigation of a super-enhancer cluster downstream of *SCN5A*, Man and colleagues have subsequently reaffirmed the importance of the *SCN5A–SCN10A* locus in the regulation of gene expression and architecture. Intriguingly, they discovered that adequate *SCN5A* expression and cardiac conduction need the interaction of all regulatory elements in the locus [113]. Future research should determine if the functional impact of these variants is associated with the dysregulation of *SCN5A* expression via the involvement of *SCN10A* as an enhancer, the function of NaV1.8 in intracardiac neurons that modulate cardiac electrical activity, or both [114].

*Sodium channel-associated*. The activity of sodium channels may be altered by circumstances other than mutations in the channel’s coding gene. In analysis assessing the impact of KV4.3 overexpression on NaV1.5 current and subsequent sodium channel availability, the results revealed that certain variables, such as the gain-of-function of the KV4.3 protein encoded by the *KCND3* gene, clearly influenced the current of the NaV1.5 protein [115]. Post-translational changes, including a malfunction in the splicing process [116] or trafficking [117], or a change in phosphorylation, methylation, or acetylation [118], might underlie abnormalities in the functionality of the channel encoded by *SCN5A* in the lack of polymorphisms in this gene. RANGRF (RAN Guanine Nucleotide Release Factor), commonly known as MOG1, modulates the development and functioning of the NaV1.5 cardiac sodium channel in individuals by augmenting the expression of NaV1.5 at the cell membrane, thereby raising sodium current density [119]. The *GPD1L* gene, which encodes a protein similar to glycerol-3-phosphate dehydrogenase-1 and is presently considered part of BrS’s diagnostic panels, modulates NaV1.5 as well [120]. The *LRRC10* gene, a transcriptional target of Nkx2.5, which modulates the ion channel proteins expressed by the *SCN5A*, *CACNA1C*, and *KCNH2* genes, is believed to be linked to BrS [121].

*Calcium channel mutations.* Considering the significance of calcium for the cardiac action potential, the key function of calcium currents in BrS is presumably underestimated. Calcium is essential to excitation–contraction coupling, connecting the electrical signal recorded by ECG that characterizes BrS to mechanical malfunction, especially ventricular fibrillation and decreased contractility, observed in BrS. Furthermore, the BrS phenotype is influenced by non-genetic variants, including a rise in vagal activity or body temperature, which are proven to affect calcium signaling. In addition, the BrS phenotype may be altered in patients by isoproterenol, which increases calcium transportation via L-type calcium channels, ryanodine receptors, and SERCA (sarcoendoplasmic reticulum calcium transport ATPase) [122]. *CACNA1C* genetic variants are responsible for around 6.6% of BrS cases, *CACNB2b* genetic variants for roughly 4.8%, and *CACNA2D1* genetic variants are rare [123,124]. *TRPM4* (Transient Receptor Potential Cation Channel Subfamily M Member 4) gene is responsible for approximately 6% of BrS cases [125], in an autosomal recessive form instead of an autosomal dominant manner [126]. According to one study, the PKP2 gene, which encodes plakophilin-2 and is located on desmosomes between intercalary discs, is related to roughly 2.5% of BrS cases [127].

*Potassium channel mutations.* Several cases of BrS have been connected to polymorphisms in potassium channel-encoding genes. The two most prevalent genes are the *ABCC9* (ATP Binding Cassette Subfamily C Member 9) and *KCNH2* (Potassium Voltage-Gated Channel Subfamily H Member 2), which account for 4–5% and 1–2% of instances, respectively. Rarely, BrS patients are linked to *HCN4*, *KCND2*, *KCND3*, and *KCNJ8* genes [71].

*Overlap Syndromes.* Considering that *SCN5A* genetic variants are present across many cardiogenetic diseases, it is not unexpected to identify an overlap between BrS and other diseases. For instance, BrS may be detected in the proband, whereas long QT syndrome, epilepsy, febrile seizures, or total bundle branch block may be observed in family members [128,129,130,131]. Multiple studies have reported an overlap between arrhythmogenic right ventricular dysplasia/cardiomyopathy (ARVD/C) and BrS [132], and the explanation might include cell–cell junctions [133]. ARVC and BrS may both develop from mutations in the connexome, with the resulting phenotype dependent on the nature of the mutation [134,135]. *PKP2* could be an essential gene in this respect since genetic variants in *PKP2* are associated with impairment of desmosomal integrity, and sodium current deficit, and can be discovered in BrS patients [127,136]. The occurrence of ARVC in BrS patients has been linked to an increased risk of arrhythmias [137]. The genetics of families with overlap syndromes should be thoroughly studied, since the genetic underpinnings may be distinct from those of families in whom BrS is the sole documented feature. This is an additional illustration of the necessity for tailored therapy and family specific BrS genetic analysis [138].

## 5. Human Induced Pluripotent Stem Cell Models

Despite the fact that animal models and primary cell lines represent the most widespread approaches for preclinical drug testing at present, there are substantial issues with them. As a potential substitute for animal models and primary cells, human induced pluripotent stem cell-derived cardiomyocytes (hiPSC-CMs) provide several benefits, including a human genome, fundamental characteristics of human CMs, and the expression of certain cardiac-specific genes [139]. To date, only several BrS models based on induced pluripotent stem cells (iPSCs) have been reported. In 2014, Cerrone et al. reported the first iPSC-based model having a rather rare mutation in *PKP2* that caused sodium current deficits [127]. In this research, hiPSC-CMs obtained from a patient with a *PKP2* deficit exhibited significantly decreased I_Na_, which was rectified by transfection of wild-type *PKP2*, but not BrS-related *PKP2*, indicating that *PKP2* genetic variants may represent a molecular substrate of BrS [127]. In 2016, Liang et al. developed patient-specific hiPSC-CMs from two patients with Brs having two genetic variants in the *SCN5A* gene and demonstrated a decrease in I_Na_ and maximum upstroke velocity of the action potentials in comparison to healthy subjects hiPSC-CMs [140]. Furthermore to *SCN5A* and *PKP2*, the Ibrahim Akin research team generated in 2019 BrS-hiPSC-CM models with genetic variants in *SCN1B* and *SCN10A* [94,141].

*RRAD* is a new gene related to Brugada syndrome susceptibility discovered by a whole-exome sequencing study [142]. In comparison to control cells from a healthy sibling, iPSC-derived cardiomyocytes obtained from the affected person had a significant drop in I_Na_ and a modest reduction in I_CaL_. In addition, over 70% of these cells had cytoskeletal abnormalities, as indicated by an abnormally spherical shape, and a decreased number of focal adhesion sites [143].The genes associated with Brugada syndrome and other inherited arrhythmia diseases are summarized in Table 1.

## 6. Arrhythmias and Sudden Cardiac Death Risk Stratification in BrS

It is widely acknowledged that individuals with symptomatic BrS have a higher probability of further arrhythmic events [13,144]. In contrast, a post-mortem analysis indicated that 72% of BrS-related SCDs happened in asymptomatic subjects. Moreover, 68% of this cohort would have been classified as low risk according to the Second Expert Consensus Conference on BrS [19]. Asymptomatic BrS patients have an annual incidence rate for arrhythmic events of between 0.5 and 1% [145,146,147]. Approximately 50% of the arrhythmic events will be represented by ventricular fibrillation despite any warning signs [148]. Even though it is challenging to quantify the progression of arrhythmic risk over the long term, considering a 1% yearly event rate, a 10% event rate at a 10-year follow-up in apparently healthy individuals is exceedingly high. Assessing the variables linked with a higher incidence of ventricular arrhythmias and SCD in BrS is a difficult undertaking [149]. Considering that implantable cardioverter-defibrillators (ICD) are linked with a 45% lifetime risk of complications [150], the option to implant them should be made sparingly. In fact, even if the development of subcutaneous ICDs may minimize the incidence of transvenous lead complications over the long term, there continues to be morbidity related to incorrect device therapies and the risk of infection with frequent generator replacements throughout time. It is consequently of the utmost significance to adequately stratify the risk of BrS patients, especially asymptomatic ones [151].

The primary challenge in individuals with a BrS type 1 ECG is to identify those who are at risk for sudden death and those who are not [144,152,153]. Several researchers have proposed identifying individuals with BrS type 1 ECG who are at risk for SD using a multiparametric clinical and instrumental strategy, including a family history of SD, syncope of presumed cardiac origin, a positive electrophysiology study, etc. Their findings indicate that individuals with several risk factors are at the greatest risk [154,155].

*Clinical Markers.* Research has shown that a history of aborted SCD is correlated with the greatest risk of subsequent arrhythmic episodes, resulting in an important prognostic significance [145,146,147]. The prevalence of arrhythmic events (sustained ventricular arrhythmia or adequate ICD therapy or SCD) was 13.5% per year in patients with a history of SCD, 3.2% per year in individuals with syncope, and 1% per year in asymptomatic patients, according to a meta-analysis [156]. Patients with a history of aborted SCD had an arrhythmia risk of 35% at 4 years [145,157], 44% at 7 years [147], and 48% at 10 years [156]. Various studies, as well as a meta-analysis, have shown that a history of syncope is related to a higher risk of subsequent arrhythmic episodes [145,146,158,159]. Priori’s team has established, nevertheless, that the correlation between syncope and transient ST segment elevation, and not a history of syncope solely, has the highest predictive value for identifying those at high risk [160]. Patients diagnosed with aborted SCD had a poor prognosis, according to Kamakura et al., whereas those asymptomatic or presenting with sole syncope had a favorable prognosis regardless of their ECG pattern [157]. Conte et al. established, nonetheless, that individuals with a history of syncope experience the same clinical course as asymptomatic patients. Specifically, throughout a long-term follow-up period of 83.8 ± 57.3 months, 11% of patients with syncope and 13% of the asymptomatic ones received adequate shocks [147]. This discrepancy may express the failure to distinguish between arrhythmic and neurally mediated syncope. Sacher et al. conducted a prospective analysis of the features of BrS patients who had suffered a syncope to establish if it could be distinguished as a syncope of arrhythmic vs. non-arrhythmic origin. Arrhythmic syncope was diagnosed in patients with no prodromal symptoms and no identified cause, who were unresponsive for no more than one minute and regained consciousness promptly. Non-arrhythmic syncope was diagnosed in patients with consciousness loss that lasted more than one minute, who had neurocardiogenic symptoms, and who did not suffer substantial physical trauma [161]. In Conte’s research, following the implantation of an ICD, 21 patients (11.9%) reported syncope. A number of five of them experienced neurally mediated syncope. In eight patients who experienced recurring syncope after ICD implantation, the percentage of ventricular pacing was less than one per cent, and no ventricular arrhythmias were observed. This may clarify why certain individuals with a history of unexplained syncope exhibit a favorable outcome [162].

Most research has shown that a family history of SCD does not predict future arrhythmic episodes in either symptomatic (3.3%) or asymptomatic (0.5%) individuals [145]. In contrast, Kamakura et al. reported that a family history of SCD occurring before age 45 is an independent risk factor for a poor outcome. Delise et al. showed that a family history of SCD could only be predictive when combined with other risk factors [154]. Regarding the more affected sex, studies have shown that the male gender has been related to a worse clinical outcome, according to a prior meta-analysis including 1545 individuals [159]. In the FINGER registry, the male gender was also linked with a lesser duration of time to the first incident, but with no statistical significance (mean incident rate per year, 3.0% for males and 0.9% for females) [145]. Additionally, in Conte’s research, men were almost three times more likely to get suitable shocks during follow-up [147]. According to the S Priori group, women with a BrS ECG pattern should not be considered a low-risk category as it was not found a significant predominance of male incidents (13%) compared to females (9%) [160].

**Figure 2:**
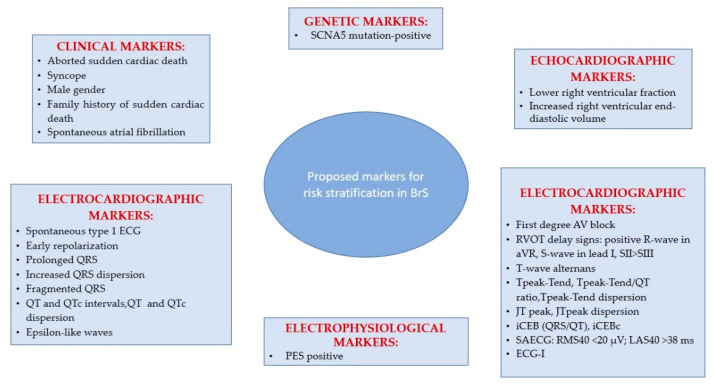
Schematic representation of the proposed markers for risk stratification in Brugada syndrome; BrS (Brugada syndrome); *SCN5A* (sodium channel protein type V subunit α gene); RVOT (right ventricle outflow tract); PES (programmed electrical stimulation); AV (atrioventricular); iCEB (index of Cardiac Electrophysiological Balance); SAECG (signal-averaged ECG); RMS40 (root mean square voltage of the QRS complex terminal 40 ms); LAS40 (duration of low amplitude signals <40 µV of the QRS complex); ECG-I (electrocardiographic imaging).

BrS patients have an increased incidence of atrial fibrillation than the overall population of the same age. In a cohort of 560 BrS patients, 9% exhibited atrial fibrillation or atrial flutter [163]. 18% of 176 BrS patients with an ICD had paroxysmal AF throughout the course of 83.8 ± 57.3 months of follow-up [147]. There is a correlation between spontaneous atrial fibrillation and an increased frequency of syncopal episodes (60.0% vs. 22.2%) and confirmed ventricular fibrillation (40.0% vs. 14.2%). Sieira et al. showed that asymptomatic BrS patients with a history of sinus node dysfunction are eight times more likely to develop subsequent arrhythmic episodes [164]. The schematic representation of the proposed markers for risk stratification in Brugada syndrome is depicted in Figure 2.

*Electrocardiographic Markers.* A meta-analysis concluded that patients with spontaneous type 1 ECG pattern had a three to four times higher risk of incidents than those with a drug-induced ECG pattern. Since they have a poor prognosis as reported in various studies, identifying BrS patients with spontaneous type 1 ECG pattern is, therefore, mandatory [165]. In addition to the spontaneous type 1 ECG pattern, a variety of other ECG characteristics have been suggested for risk stratification, among which QRS fragmentation looks to be the most compelling of all ECG indicators. The fragmented QRS complex, which reflects a rise in conduction dispersion, might cause a unidirectional block [166,167,168], while broad QRS, which reflects a decrease in conduction velocity, will lower the excitation wavelength [169]. Both are risk factors for re-entrant arrhythmias. One study has shown that QRS-fragmentation is more prevalent in BrS patients with VF (85%) and syncope (50%) than in those without symptoms (34%) [170], an aspect that was validated by the PRELUDE trial, which demonstrated that QRS-fragmentation is an independent predictor of subsequent arrhythmias [146]. Furthermore, Tokioka et al. have established that the occurrence of QRS-fragmentation increases five times the risk of arrhythmic episodes [171].

Other ECG markers independently linked to SCD or adequate ICD therapy are first-degree atrioventricular block [172], prolonged QRS duration in leads II, V2, and V6 [173,174], and a prolonged QTc interval >460 ms in lead V2 [175]. Based on the results of preclinical research, it was determined that conduction anomalies must be included in risk indicators to improve their predictive ability [176,177]. For instance, the index of Cardiac Electrophysiological Balance (iCEB), provided by QT/QRS, is a substitute marker of λ, and its application has resulted in more accurate risk stratification [178,179].

Multiple ECG repolarization indicators for stratifying arrhythmic risk in the BrS population have been explored [180]. Tpeak–Tend interval, considered a measure of transmural scattering of repolarization [175], has been associated with malignant ventricular arrhythmias, although research on swine models revealed that it is a measure of global dispersion of repolarization rather than transmural [181]. The Tpeak–Tend interval through lead V1 to lead V4, the threshold value and the distribution throughout all precordial leads were substantially greater in symptomatic patients (aborted SCD, proper ICD treatment, syncope) than in asymptomatic patients. A value of the Tpeak–Tend interval of >100 ms was independently associated with arrhythmic episodes [172].

A negative T-wave in lead V1 has also been linked to a worse prognosis [182]. Furthermore, the development of T-wave alternans following treatment with pilsicainide was predictive of spontaneous VF [183]. In a comprehensive study of 166 BrS patients who underwent exercise testing, ST-segment elevation was reported in 57% of individuals with BrS, with ST elevation appearing during early recovery in 93 patients, while in two patients it appeared during the effort phase of the exercise and in five individuals the BrS ECG pattern was revealed by the exercise. Ventricular arrhythmias, including ventricular tachycardia and numerous premature ventricular contractions, were developed in three patients, all of them happening during the first phase of recovery and they resolved spontaneously [184]. Makimoto et al. found that ST-segment elevation during early recovery was distinctive in BrS patients and was substantially related to a greater cardiac event rate, particularly in patients with a prior episode of syncope or in asymptomatic individuals [185]. In contrast, Amin et al. were unable to demonstrate substantial differences among symptomatic and asymptomatic BrS patients in terms of ECG characteristics and their alterations throughout the exercise [186]. It has been found that an inferolateral early repolarization pattern is associated with arrhythmic episodes [187,188]. Tokioka et al. have demonstrated that the coupling of QRS-fragmentation and early repolarization pattern facilitates the assessment of individuals at high risk [171]. In prior research involving 290 persons with BrS, an early repolarization pattern characterized as notched or slurred J-point elevation primarily in lateral leads was found in 12% of the participants (35 individuals). Nonetheless, the appearance of an early repolarization pattern was not related to arrhythmic episodes throughout the course of this research [189]. After the follow-up, the aVR sign, defined as R wave ≥ 0.3 mV or R/q ≥ 0.75 in lead aVR, was linked to arrhythmic episodes [190].

The signal-averaged ECG (SAECG) uses three orthogonal leads aligned in the X, Y, and Z planes to quantify cardiac electrical activity and the software algorithms reduce noise and expose low-voltage signals [191]. Late potentials (LPs) are low voltage indicators of delayed conduction and have been associated with a higher risk for malignant cardiac events in BrS patients meeting the following conditions: RMS40 (root mean square voltage of the QRS complex terminal 40 ms) <20 µV and LAS40 (duration of low amplitude signals <40 µV of the QRS complex) >38 ms [192]. Ajiro et al. demonstrated that symptomatic individuals with BrS had considerably lower RMS40, greater LAS40, and longer filtered QRS duration than asymptomatic individuals [193]. Daily variations in ECG and SAECG features might be used to differentiate across BrS patients at high and low risk [194].

Electrocardiographic imaging (ECG-I) has shown significant conduction delays in the RVOT, and the administration of ajmaline exacerbates these abnormalities [51]. The extent and/or region of delay could be an additional valuable indicator for predicting risk, an ECG-I strategy to risk prediction has been suggested in an exercise stress-based pilot research [195].

*Echocardiographic Markers.* In one study, two echocardiographic parameters, a lower right ventricular ejection fraction and an increased right ventricular end-diastolic volume were linked with a history of syncope or SCD [196]. Applying tissue velocity imaging, Van Malderen et al. found a correlation between a history of malignant incidents and a prolonged right ventricular ejection delay [197].

*Genetic Markers.* Even though a genetic mutation in the *SCN5A* gene was not related to an increased risk of subsequent arrhythmic episodes in several prior studies [145,146,147] suggesting that genetic testing was a valuable diagnostic indicator but not effective for risk stratification, recent studies propose the *SCN5A* genetic status as a prognostic indicator for BrS patients [198,199]. Specifically, mutation carriers of *SCN5A* had more significant epicardial electrical anomalies and a more severe clinical phenotype. At least in a subset of patients, the mutant *SCN5A* gene works more as a phenotypic modulator than a true Mendelian dominant cause of the exhibited phenotype, thereby putting into doubt the autosomal dominant inheritance of BrS. This also holds for variations of “unknown significance” (VUS), which are often considered “benign”, but several of these VUS are subsequently categorized as pathogenic. Given that the oligogenic hypothesis is expected to be adopted in the near future, it is anticipated that additional VUS, particularly those in the *SCN5A* gene, will eventually be proven to be pathogenic [138].

*Electrophysiological Markers*. There are contradictory findings on the prognostic significance of electrophysiology studies (EPS) in asymptomatic BrS patients. Programmed ventricular stimulation (PVS) is an invasive electrophysiological procedure that involves direct stimulation of the RV apex and RVOT, with 1–3 premature extra stimuli following a specific number of impulses with a predetermined cycle length [200]. In the multinational SABRUS (Survey on Arrhythmic Events in Brugada Syndrome) research, VF inducibility throughout PVS was linked with a comparable time to the onset of the first arrhythmic episode as individuals with an *SCN5A* mutation and a family history of SCD, but longer compared to those with spontaneous type 1 ECG or syncope [201]. In contrast, in the PRELUDE (PRogrammed ELectrical stimUlation preDictive valuE) [146] and FINGER (France, Italy, Netherlands, Germany) [145] registries, a negative EPS was not related to a lower risk of an arrhythmic episode. In addition, there is controversy around the positive predictive value of EPS, since numerous studies have demonstrated that VT/VF inducibility is predictive of future arrhythmic episodes [202]; however, other research [145,147] does not validate the application of EPS for risk stratification. In one study, patients with VF inducibility had an event-related hazard ratio of 8.3. The event-free survival at one year was 99.0% for the non-inducible group and 89.0% for the inducible one and 100.0% for the asymptomatic patients without EPS inducibility [164].

Faucher et al. conducted a meta-analysis of 13 studies examining the prognostic significance of EPS in BrS patients based on their clinical manifestation. VF inducibility was linked with a nonsignificant increased risk of arrhythmic episodes throughout follow-up across the entire cohort of BrS patients. In contrast, the induction of sustained ventricular arrhythmia was substantially and uniformly related to a higher risk of arrhythmic episodes throughout follow-up in patients who suffered syncope [156]. Sroubek et al. conducted a meta-analysis to assess the significance of PVS in the risk stratification of BrS patients. Inducibility of VT or VF was related to a two- to three-times higher risk of malignant cardiac events. Nonetheless, patients with no inducible arrhythmias also suffered ventricular episodes throughout follow-up [203]. PVS is presently classed as a class IIb recommendation, where it may be applied for risk stratification in asymptomatic individuals with a spontaneous type 1 ECG [204].

*Multiparametric Risk Stratification Scores.* The link between these indicators and the value of their combination has not been thoroughly investigated. The Shanghai [24] and Sieira [205] scores have been recently postulated, to identify Brugada syndrome and to stratify the risk of SD in individuals with a BrS type 1 ECG. Overall, a gain in the cumulative score was related to a rise in the number of patients suffering life-threatening arrhythmias, showing that patients with a higher score exhibited an increased risk for VT/VF. Most of the patients with a high score had clinical manifestations, a family history of sudden death, and positive genetic testing. A score of 3.50 was the deciding factor with nearly 50% of patients classified into this group. Combining a spontaneous type 1 ECG pattern with either a strong clinical or family history, but not both, yielded a score of 4 to 5 points. The coupling of a spontaneous and/or drug-induced type 1 ECG with a major clinical and family history was assigned a score of ≥5.50. The event-free survival curves indicated that patients with 3.5 points had a moderate risk for VT/VF, those with 4 to 5 points had a high risk, and those with ≥5.5 points had the greatest risk. Thus, the prognosis was reliant on the cumulative score, and the incidence rate increased as the score ascended [24]. Probst et al. [206] analyzed a considerably wide group (1613 patients) across an extended period (mean 6.5 years), with patients divided into two groups: 477 with an ICD and 1136 without. In the first case, the outcome was the detection of rapid ventricular arrhythmias by the device or the delivery of adequate shocks. In the second case, the outcome was SD or aborted SD. In a multicenter study [207], the significance of 16 hypothesized risk variables for VA/SCD in BrS was assessed in one of the biggest BrS cohorts yet reported. PAR syncope and a spontaneous type 1 Brugada ECG pattern continue to be independent predictors of VA/SCD, consistent with the results of a significant number of BrS research. In this study, the identification of ER and a type 1 Brugada ECG pattern in peripheral leads, which had initially only been evaluated in a small number of studies, was discovered to be independent predictors of VA/SCD.

## 7. Conclusions

Current knowledge of the genetic and molecular pathogenesis of Brugada syndrome is constrained by a lack of a gold standard, being a challenging pathology regarding the diagnostic, arrhythmia risk stratification and management. Nonetheless, extensive and in-depth research about the genetic and molecular underpinnings continues to provide new perspectives. This encompasses the growing recognition of an underlying structural substrate’s importance. For the attending physician, identifying BrS patients at high risk of arrhythmia and SCD is a difficult task. Given the paucity of data, it is of the utmost importance to stratify risk using all applicable tools/modalities that have proven prognostic value. Thus, the diagnostic result may be improved if the present instruments are used adequately. Multiparametric scores tend to enhance risk stratification, while single risk variables have limited predictive relevance. Therefore, the elaboration and validation of a model risk score that integrates various clinical, genetic, electrocardiographic, and electrophysiologic markers, as well as environmental factors may enhance the prediction of arrhythmia and sudden cardiac death and could improve therapy management. Focusing on technologies that can enhance iPSC-CM maturity and achieve desirable engineered cardiac tissues, as well as standardizing experimental procedures and conducting more in-depth (functional) analyses of the obtained iPSC-CM models, may enable bridging the gap across model and clinical practice.

## Figures and Tables

**Table 1:** Genes associated with Brugada syndrome (BrS) and other inherited arrhythmia diseases [124].

INHERITED CARDIAC DISEASES	GENES ASSOCIATED
LQT	*SCN5A* SCN1B CACNA1C CACNB2b CACNA2D1
SQT	CACNA1C CACNB2b CACNA2D1
SSS/PCCD	*SCN5A* SCN1B SCN2B SCN3B *SCN10A* RANGRF GPD1-L SLMAP
ERS	CACNA1C CACNB2b CACNA2D1 ABCC9 KCND3 KCNE3 KCNE5 KCNJ8 KCNH2 HCN4
ARVC	PKP2 TRPM4 HEY2

ARVC (arrhythmogenic right ventricular cardiomyopathy); ERS (early repolarization syndrome); LQT (long-QT syndrome); PCCD (progressive cardiac conduction defect); SQT (short-QT syndrome); SSS (sick sinus syndrome).

## Data Availability

Not applicable.
